# Adverse Effects of Carbetocin versus Oxytocin in the Prevention of Postpartum Haemorrhage after Caesarean Section: A Randomized Controlled Trial

**DOI:** 10.1155/2018/1374150

**Published:** 2018-01-02

**Authors:** D. Mannaerts, L. Van der Veeken, H. Coppejans, Y. Jacquemyn

**Affiliations:** ^1^Department of Obstetrics and Gynaecology, Antwerp University Hospital (UZA), Edegem, Belgium; ^2^Research Group ASTARC (Antwerp Surgical Training, Anatomy and Research Centre), University of Antwerp (UA), Antwerp, Belgium; ^3^Department of Anaesthesiology, Antwerp University Hospital (UZA), Edegem, Belgium

## Abstract

**Purpose:**

To compare the incidence of nausea, vomiting, and arterial hypotension between carbetocin and oxytocin to prevent haemorrhage after caesarean section (CS).

**Methods:**

A randomized controlled trial in term pregnant women undergoing planned CS. Groups were randomized to carbetocin or oxytocin. Blood pressure (BP), heart rate, presence of nausea/vomitus, and need for vasopressors were evaluated throughout surgery. Preoperative and postoperative haemoglobin and haematocrit levels were compared.

**Results:**

Fifty-eight women were randomized (carbetocin *n* = 32; oxytocin *n* = 26). Both medications had hypotensive effect, difference in BP for carbetocin versus oxytocin: systolic (14.4 ± 2.4 mmHg versus 8.5 ± 1.8 mmHg); diastolic (7.8 ± 1.6 mmHg versus 8.9 ± 3.0 mmHg) without significant difference between the drugs (*p* = 0.1 and *p* = 0.7). Both groups had similar needs for vasopressors. The presence of nausea was not rare, but the difference was not statistically significant (*p* = 0.4). Average blood loss was slightly lower in the carbetocin group but not statistically significant (*p* = 0.8).

**Conclusion:**

In planned CS, a possible clinical significant lower incidence of nausea after carbetocin was noted but this was not statistically significant. There were no differences regarding BP, heart rate, the need for vasopressor, and blood loss. The study was registered in the International Journal of Clinical Trials (ISRCTN 95504420, 2/2017).

## 1. Introduction

Postpartum haemorrhage (PPH) constitutes a major cause of maternal morbidity and mortality [1] and complicates approximately 6% of all deliveries [2]. The most frequent cause of PPH is uterine atony, contributing up to 80% of cases [3]. Among others, caesarean section (CS) is a well-known risk factor for PPH and it is advised to systematically administer uterotonic agents immediately after extraction of the foetus [4].

Currently oxytocin is most frequently used as agent of first choice after caesarean section. Due to its short half-life (4 to 10 minutes), it requires continuous or frequently repeated administration. More recently carbetocin has been developed as a long acting oxytocin agonist and when administered it results in a sustained uterine contraction. In a systematic review and meta-analysis of randomized controlled trials, carbetocin is associated with reduced need for additional uterotonic agents, but no differences are noted for PPH, severe PPH, mean estimated blood loss, or adverse effects [5]. Different side effects including nausea, vomiting, or arterial hypotension eventually resulting in dizziness or even syncope have only been studied as secondary endpoints of randomized controlled trials [6, 7]. Since carbetocin is a modified version of oxytocin, it should be expected that possible side effects might be similar. Hypotension, an important haemodynamic side-effect, has been described using both oxytocin and carbetocin [8, 9]. When comparing carbetocin with low dose oxytocin, haemodynamic side effects seem to be comparable in both groups. No difference in hypotension has been noted between different doses from 20 to 100 *μ*g of carbetocin and generally hypotension is noted in 40 to 55% [10, 11].

In this trial, we aim to compare the most frequent adverse effects of both carbetocin and oxytocin, that is, nausea, vomiting, and flushing during primary uncomplicated caesarean section and the haemodynamic effect. We hypothesize that both drugs will have comparable effects.

## 2. Methods

### 2.1. Study Design

The study protocol has been published previously [12]. Briefly, in this single centre, double blind, randomized trial, two active intervention arms are compared, one with carbetocin and the other with oxytocin. Participants are randomly assigned following simple randomization procedure in 1 : 1 ratio to one of the two treatment groups. A computer-generated randomization list was generated using SPSS21. Medication was prepared by a midwife not treating the patient to make sure that patient, gynaecologist, anaesthesiologist, and midwife clinically in charge of the patient are blinded for the medication. The study took place at Antwerp University Hospital (UZA), Belgium. Participants were recruited at the delivery ward at the moment they arrived at the hospital for planned caesarean delivery. Women, with singleton pregnancies undergoing a planned caesarean section at term (≥37 weeks) under combined spinal/epidural anaesthesia were all included. Women with medical conditions potentially influencing outcome measures (nausea, vomitus, and hypotension) were excluded: diabetes, preexisting hypertension, preeclampsia, gestational hypertension, and known gastrointestinal diseases. The Research and Ethics Committee of the Antwerp University Hospital approved the study protocol (Belgian number: D300201110299), and written informed consent was obtained from all subjects. The study was registered in the International Journal of Clinical Trials as ISRCTN 95504420 (February 2017) [12].

### 2.2. Intervention

The control group received the standard dose of oxytocin (Syntocinon, Sigma-Tau, Rome, Italy) as used in our hospital, 5 IU (International Units) oxytocin in 10 ml NaCl 0.9% over 3 minutes followed by 10 IU oxytocin in 1000 ml of crystalloid (Plasma-Lyte®, Baxter SA, Belgium) over 24 hours. The study group received 100 *μ*g of carbetocin (Pabal®, Ferring NV, Aalst, Belgium) in a single dose in 10 ml of NaCl 0.9% over 3 minutes followed by 1000 ml of crystalloid (Plasma-Lyte, Baxter SA, Belgium) over 24 hours. All patients underwent surgery using the same standardized Joel-Cohen technique for caesarean section as generally used in our hospital. Patients received phenylephrine or ephedrine in bolus (depending on heart rate) when systolic blood pressure (BP) dropped more than 10% compared to baseline or systolic BP < 100 mmHg.

### 2.3. Outcomes

#### 2.3.1. Primary Outcomes

Nausea and vomiting were evaluated every three minutes with the following scale: 0 = no nausea and no vomitus; 1 = mild nausea and no vomiting; 2 = moderate nausea and no vomiting; 3 = severe nausea and vomiting. Flushing was noted as present or absent. Heart rate and BP are measured every three minutes starting from administration of anaesthesia until the end of surgery.

#### 2.3.2. Secondary Outcomes

As secondary outcomes, the difference in haemoglobin and haematocrit taken 24 to 2 hours before the intervention and 48 hours after the intervention was registered. This serves as a substitute for the total amount of blood lost and postpartum haemorrhage. Furthermore, need for additional uterotonics was noted. Secondary to the haemodynamic effect, the need for vasopressive medication was documented.

### 2.4. Statistical Analysis

Analysis is conducted according to intention to treat and per protocol. We calculated that 150 patients per group would provide 80% power and a statistical significance of 0.05 to detect a 15% to 5% decrease in the incidence of nausea and vomiting among the treatment groups; we considered this difference to be clinically relevant. Dichotomic variables including nausea and vomiting are compared by Chi-squared test or Fisher's exact test as appropriate. For continuous variables, Student's *t*-test was used for normally distributed data. For all tests, significance was accepted at *p* < 0.05.

## 3. Results

### 3.1. Patient Characteristics

**Figure 1:**
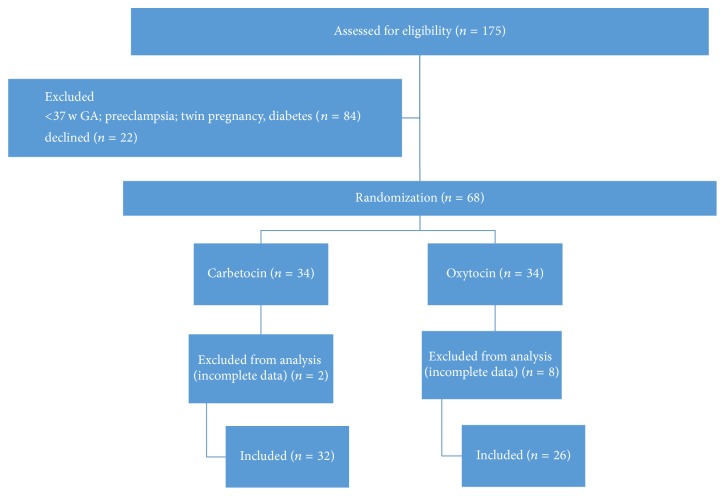
Flow chart of inclusions.

Sixty-eight women were enrolled and equally randomized in the two treatment groups. Ten women were excluded from analysis (eight in the oxytocin and two in the carbetocin group) because of incomplete data (Figure 1). The trial was stopped prematurely before the planned inclusions were completed because of slow inclusion (due to lower numbers of planned term caesareans in low risk patients in our institution), since we feared the influence of changes in anaesthesiological protocols. Patient characteristics of both groups were similar, demonstrating correct randomization (Table 1). Gestational age was in all patients 38 to 40 weeks.

### 3.2. Primary Outcomes

All patients received medication as per randomization list, so intention to treat and per protocol analysis are identical. Regarding primary outcomes, we found no significant difference between groups. Side effects in both groups were equal: 23% with carbetocin versus 22% with oxytocin (Table 2). Nausea was present in 2/32 (6%) and 4/26 patients (15%) for carbetocin and oxytocin, respectively; there was no significant difference (*p* = 0.256). Flushing could be seen in 4/32 (13%) and 2/26 (7%) patients, respectively (*p* = 0.550). Only one patient in the oxytocin group required an antiemetic agent.

**Figure 2:**
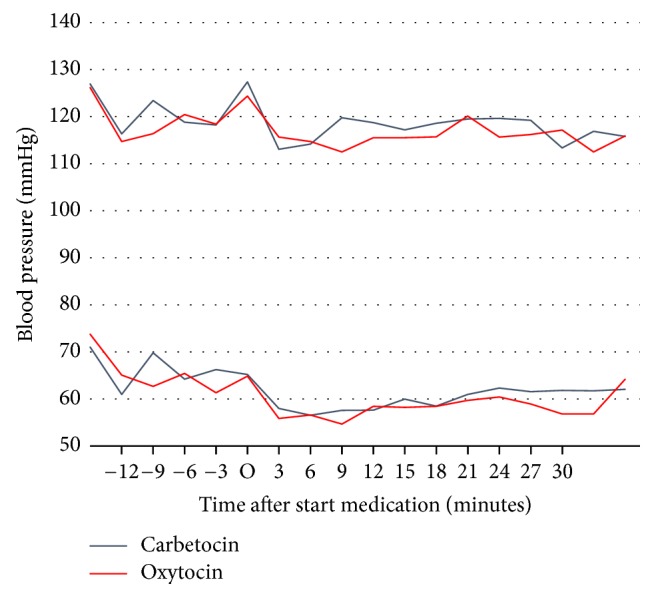
Maternal systolic and diastolic blood pressure from start anaesthesia until the end of the procedure; time point O = start medication; data are displayed as means.

**Figure 3:**
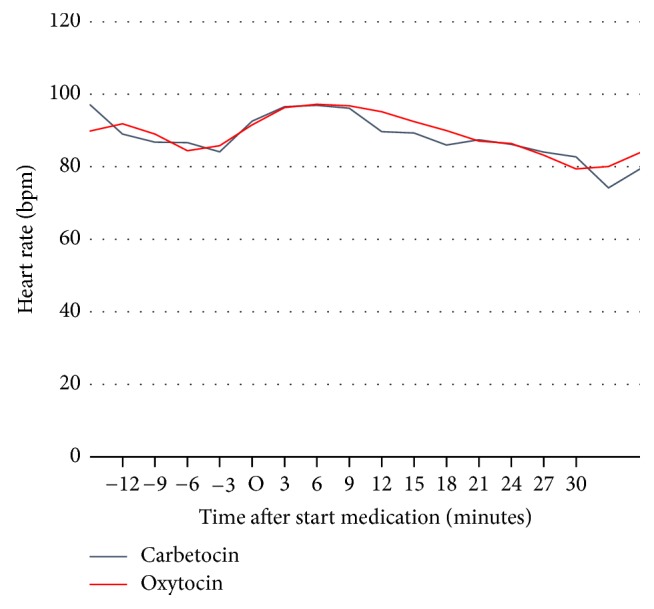
Maternal heart rate from start anaesthesia until the end of the procedure; time point O = start medication; data are displayed as means.

Figures 2 and 3 show the evolution of systolic pressure, diastolic pressure, and heart rate in time, measured every 3 minutes. Both groups had similar BP and heart rate preoperatively. The haemodynamic effects of both oxytocin and carbetocin consist of vasodilatation and result in hypotension. This is visible after three minutes and remains stable for the entire procedure. The mean drop in systolic BP after 3 minutes was 14.4 mmHg (95% CI 9.5–19.3) for carbetocin and 8.6 mmHg (95% CI 4.8–12.4) for oxytocin and diastolic pressure dropped to 7.8 mmHg (95% CI 4.5–11.1) versus 8.9 mmHg (95% CI 2.8–15.0), respectively. Between groups, there was no significant difference in BP after administration, nor did this change after 6 or more minutes.

Mean heart rate did not change after carbetocin or oxytocin treatment.

### 3.3. Secondary Outcomes

The use of vasoactive medications (phenylephrine or ephedrine) was necessary to maintain BP at an acceptable level in 25% of carbetocin patients versus 23% in the oxytocin group, which did not differ significantly (*p* = 1.0). Patients who required vasoconstrictive medication received in the majority of cases multiple doses to maintain adequate BP.

Mean ΔHb was slightly higher in the oxytocin group 1.50 g/dL versus 1.45 g/dL but the difference was not significant (*p* = 0.8), nor was the difference in haematocrit: 4.08 versus 3.80 (*p* = 0.7) for oxytocin and carbetocin, respectively. Need for additional uterotonics preoperatively did not occur in the carbetocin group, while two patients who received oxytocin needed additional carboprost (*p* = 0.2).

## 4. Discussion

Up until now, only a couple of trials have been conducted investigating the difference in haemodynamic effects, that is, effect on BP and heart rate, between oxytocin and carbetocin [6]. To the best of our knowledge, no previous study observed nausea, vomiting, and flushing as a primary outcome. We found nausea and vomitus to be present in a clinically relevant percentage of patients, 15% and 6% in oxytocin and carbetocin, respectively. Although not statically different, such difference may be clinically relevant. Furthermore, we did not see any differences regarding BP/heart rate and need for vasopressors. Finally, we noted that carbetocin and oxytocin result in equal postoperative versus preoperative haemoglobin differences.

Caesarean section remains a risk factor for PPH [1, 2]. The prophylactic use of uterotonics reduces mean blood loss and therefore maternal morbidity and mortality. Although oxytocin has long been the product of first choice, carbetocin has found its place in modern obstetrics. Up until now, the best product for the prevention remains subject for discussion [5]. Both products are believed to have a similar mechanism of action; that is, oxytocin and carbetocin bind to the same receptor [13]. Oxytocin is a receptor agonist and carbetocin is a long working variant. A study by Cole et al. compared the in vitro effect of oxytocin and carbetocin on the contractility of myometrium samples obtained during elective caesarean section and found the former to be more effective [14]. Concerning the in vivo effect, multiple studies have compared oxytocin and carbetocin in primary caesarean section but no differences could be seen in effectiveness. Regarding the adverse effects only a few studies have been conducted.

Nausea, vomitus and flushing are the most frequent adverse effects encountered when carbetocin or oxytocin is used for the prevention of PPH. Moertl et al. [6] described in a randomized trial and the effects on BP and heart rate. As a secondary outcome, they mentioned that nausea, vomitus, flushing, and headache were most the common adverse effects and they seemed equal between carbetocin and oxytocin. In our study, we confirmed that these side effects are equal between the two products. Nausea was slightly more frequent in the oxytocin but adversely flushing was seen more frequent in the carbetocin group. Overall side effects remained rare in both groups.

Both carbetocin and oxytocin are known to cause hypotension, certainly when administered in high doses for the prevention of PPH. In our centre, we use a lower dose of oxytocin as uterine contractility is sufficient after 3 IU [15], and the hypotensive effect could be diminished. As far as we know, in most centres in Belgium, a standard dose of 10 IU (=1 ampulla) of oxytocin is given after caesarean section. It is possible that with such a high dose of oxytocin difference in nausea and vomitus would be still higher, resulting in a statistically significant lower incidence in the carbetocin group. We do not think a trial should be set up for this, as the “1-ampulla” dose is proven to be more than the necessary amount for the prevention of postpartum haemorrhage after caesarean section. We found that, even with the lower dose administered, oxytocin has a hypotensive effect and this was comparable to that of carbetocin. After initial drop in BP, the majority of patients remained stable until the end of the procedure. These haemodynamic effects of carbetocin and oxytocin we found were comparable to those found by Moertl et al. [6] and Larciprete et al. [9]. They described the same drop in BP immediately after administration and a recovery phase afterwards. If we look at our graphs we see the same trend. Concerning the minimum effective dose of carbetocin, the discussion remains open. A recent study by Khan et al. found that carbetocin appears to be equally effective in a dose that is less than one-fifth of the currently recommended dose of 100 *μ*g. In the same paper, a lower incidence of hypotension as a secondary outcome was reported [16]. Although most studies report an effective dose of 100 *μ*g, in the future larger comparative studies should be set up with lower doses of carbetocin and adverse effects as primary outcomes [10].

As previous found by Jin et al. [5], we neither could see a difference in efficacy between oxytocin and carbetocin regarding mean blood loss. In the past, several studies looked at the most effective agent for prevention of PPH. Although need for additional uterotonics was in favour of carbetocin, none of these studies could identify a significant difference in estimated blood loss, need for transfusion, or mean drop in haemoglobin. Our study confirmed these findings, although this was not the primary endpoint. Difference in haemoglobin was lower for carbetocin, but this was neither statistical nor clinically significant. If we look at the need for additional uterotonics or need for blood transfusion, no differences could be found.

The strength of our study is the homogenous group, without other confounding factors influencing nausea and vomitus such as concomitant medication, previous labour, or differences in surgical technique. Limitations of this study are multiple. A major point of weakness is that we stopped the trial prematurely, which means that we cannot exclude that more patients could have been included and that a significant difference could still appear. The randomized groups were too small to reach the number calculated in our power analysis. It was decided to stop the trial after two years because of lack of inclusions. This was mainly due to the fact that planned caesarean sections in term women are extremely rare in our institution, most being for breech or repeat caesarean sections (Table 1).

In these setting, we only included patients admitted for planned caesarean; in an emergency setting, difference in haemodynamic effect could be pointed out and differences could become clearer. Adverse effects as nausea and vomitus could change in a setting with a nonsober patient. The nausea scale we used in this study is a nonvalidated scale, which has its limitations in statistical power. On the other hand, our study is the first that primary investigates the differences in adverse effects in healthy subjects, which is a clinically relevant outcome measure. The population was randomized correct, which gave similar group characteristics. BP measurements were automatic, so there was no interobserver variation.

## 5. Conclusion

We conclude that oxytocin and carbetocin have a similar effect on nausea and vomiting; if there is any difference it would be that carbetocin probably results in less nausea and vomiting, which may be clinically relevant although the difference did not reach statistical significance due to the lack of sufficient power in this study. Both products have similar influence on BP, heart rate, the need for vasopressor, and blood loss.

## Figures and Tables

**Table 1:** Patient characteristics.

	Oxytocin *N* = 26	Carbetocin *N* = 32	*p*
Age	29,9 ± 4.1	31,3 ± 4,3	0.2
Primiparous	7 (27%)	7 (22%)	NA
Indication for caesarean: repeat	16 (62%)	22 (69%)	NA
Indication for caesarean: breech	6 (23%)	6 (19%)	NA
Preoperative Hb (g/dL,)	11,7 ± 1,3	11,8 ± 1,3	0.8
Preoperative Hct (%)	33,9 ± 3,1	34,1 ± 3,4	0.7
Systolic blood pressure (mmHg) last value before spinal/epidural	128 ± 13,3	129 ± 16,5	0.1
Diastolic blood pressure (mmHg) last value before spinal/epidural	76 ± 9,1	81 ± 9,3	0.3
Heart rate (beats per minute) last value before spinal/epidural	94 ± 14,0	90 ± 12,4	0.3

All values are mean ± standard deviation. NA: not applicable.

**Table 2:** Adverse effects.

	Oxytocin *N* = 26 *N* (%)	Carbetocin *N* = 32 *N* (%)	*p*
Nausea	4 (15.3)	2 (6.2)	0,3
Flushing	2 (7.7)	4 (12.5)	0,6
Need for vasopressors	6 (23.1)	8 (25)	1,0
Need for other uterotonic	2 (7.7)	0 (0)	0,2
Need for anti-emetics	1 (3.8)	0 (0)	0.5
ΔHb (g/dL)	1,50 ± 0,9	1,45 ± 1,1	0,8
ΔHct (%)	4,08 ± 2,8	3,80 ± 3,0	0,7

ΔHb = (preoperative haemoglobin) − (haemoglobin 48 h after caesarean section) ± standard deviation; ΔHct = (preoperative haematocrit) − (haematocrit 48 h after caesarean section) ± standard deviation.
